# Adaptive evolution of an industrial strain of *Saccharomyces cerevisiae* for combined tolerance to inhibitors and temperature

**DOI:** 10.1186/1754-6834-6-151

**Published:** 2013-10-20

**Authors:** Valeria Wallace-Salinas, Marie F Gorwa-Grauslund

**Affiliations:** 1 Applied Microbiology, Department of Chemistry, Lund University, PO Box 124, SE-221 00 Lund, Sweden

**Keywords:** *Saccharomyces cerevisiae*, Evolution, Thermotolerance, Hydrolysate, Inhibitors, Ethanol

## Abstract

**Introduction:**

Development of industrial yeast strains with high tolerance towards the inhibitors released during biomass pretreatment is critical for bioethanol production. Combining this trait with increased thermotolerance would result in a more efficient production via Simultaneous Saccharification and Fermentation (SSF) as well as reduced cooling costs. The aim of the present work was to develop a yeast strain combining these traits.

**Results:**

Using a long-term adaptation strategy a stable *Saccharomyces cerevisiae* isolate (ISO12) was evolved from the industrial strain Ethanol Red (ER). ISO12, contrary to the parental strain, is capable of growing and fermenting the liquid fraction of non-detoxified spruce hydrolysate at 39°C with an ethanol yield of 0.38 g ethanol . g hexoses^-1^. In contrast with previous studies, the superior phenotype of ISO12 does not rely on higher reductase activities for furaldehyde inhibitor conversion, but rather on a higher thermotolerance. ISO12 shows a higher capacity to ferment hydrolysate at 39°C and higher viability during heat-shock at 52°C than ER. In the absence of inhibitors, however, both ER and ISO12 displayed similar growth phenotype at 39°C.

**Conclusions:**

The evolved isolate ISO12 shows a superior phenotype than the parental strain ER when both stresses, temperature and inhibition by hydrolysate-derived compounds, are applied together. The results suggest that the presence of inhibitors depress the maximum temperature permissible for growth to a value below 39°C. As a result of the adaptation process and acquired improved thermotolerance, ISO12 is able to overcome this synergistic effect. Robust strains, such as ISO12, are interesting candidates for second generation ethanol production by SSF, as well as in tropical countries where fermentations at higher temperature can positively impact the production costs.

## Background

Baker’s yeast *Saccharomyces cerevisiae* is the preferred microorganism for large scale ethanol production due to its high ethanol yield and productivity and general robustness. However, as strategies are put in place to generate larger and cheaper ethanol volumes worldwide, *S. cerevisiae* is further challenged with new process requirements. For example, the use of lignocellulosic feedstock impact yeast fermentation via the formation of inhibitory substances such as weak acids, furaldehydes and phenolics during the biomass pre-treatment, which negatively affect strain performance [1]. Additionally, yeasts with higher thermotolerance are needed in a simultaneous saccharification and fermentation process (SSF), since the current compromise between the optimal fermentation and saccharification temperatures (30-35°C and ca. 55°C respectively) significantly limits the rate of the enzymatic hydrolysis [2,3]. Also, the use of more thermotolerant yeasts would enable a reduction of operational costs, especially in tropical countries [4].

A significant number of approaches aiming at minimizing the effects of hydrolysate-derived compounds on the performance of the yeast have been evaluated (for a recent review see [5]). For example, S*. cerevisiae* yeast strains with increased tolerance towards hydrolysate-derived inhibitors have been obtained by genetic engineering [6-10] and evolutionary engineering strategies [11-15] the latter of which has also been reviewed recently [16]. Similarly, the isolation and development of thermotolerant yeasts has included the screening of collections and new habitats [17,18] as well as targeted engineering approaches [19,20].

Achieving fermentations of lignocellulosic hydrolysate-based media at high temperatures would require the development of strains with combined resistance traits. However, the number of studies in which both conditions are considered together is limited [21,22]. The aim of the current study was to increase the robustness of an industrial *S. cerevisiae* strain by improving the tolerance towards both high temperature and hydrolysate-inhibitors through an evolutionary engineering strategy. Here we describe the generation and characterization of a stable isolate, capable of growing and fermenting non-detoxified spruce hydrolysate at 39°C with an ethanol yield of 0.38 g ethanol. g hexoses^-1^.

## Results

### Evaluation of the innate strain tolerance to lignocellulosic hydrolysate

The robust industrial strain Ethanol Red (ER) was chosen for the evolution experiment in order to select for genetic changes that are not linked to the generally poorer performances of laboratory strains. ER was first evaluated for aerobic growth in the presence of increasing concentrations of undetoxified spruce hydrolysate. The experiments were performed at 35°C, which is very close to the reported optimum fermentation temperature of ER [23]. No growth was observed in media with 80% (v/v) hydrolysate or more after 140 h. In 50% hydrolysate, ER grew after a long lag phase and the growth rate was approximately 40% lower than the rate obtained in mineral medium without hydrolysate (Table 1). Hence, medium with 50% hydrolysate was chosen for the adaptation process as it would impose a significant pressure on the cells without completely abolishing growth.

**Table 1:** Growth parameters of Ethanol Red in increasing concentrations of spruce hydrolysate at 35°C

**% (v/v) of spruce hydrolysate**	**Lag phase (approx. values)**	**Specific growth rate (1/h)**
0	1.5 h	0.495 ± 0.05
25	3.5 h	0.375 ± 0.05
50	25 h	0.285 ± 0.05
80	No growth after 140 h	

Experiments have been performed in biological duplicates.

### Strain adaptation

It has been shown that two of the most relevant inhibitors of lignocellulosic hydrolysates, the furaldehyde derivatives furfural and 5-(hydroxymethyl) furfural (HMF), are reduced by yeast to their corresponding and less inhibitory alcohol forms [24,25]. In the case of furfural, the conversion mainly takes place during the lag phase, while HMF-reduction occurs while the yeast is growing and sugars are being consumed [13,26]. Consequently, the adaptation process was performed as repeated batch cultures by pumping broth out of the bioreactor and replacing it with fresh medium when the culture reached mid-log phase. By replacing the medium during the exponential growth phase, the population in the bioreactor would be forced to adapt to a new load of inhibitors when the state of the cells generally gives a low tolerance towards stress [27,28]. This was expected to aid the development of inhibitor tolerance as the yeast would regularly be exposed to non-reduced forms of the furaldehyde inhibitors (together with “fresh”/ non-metabolized forms of the other types of inhibitors found in the hydrolysate).

**Figure 1:**
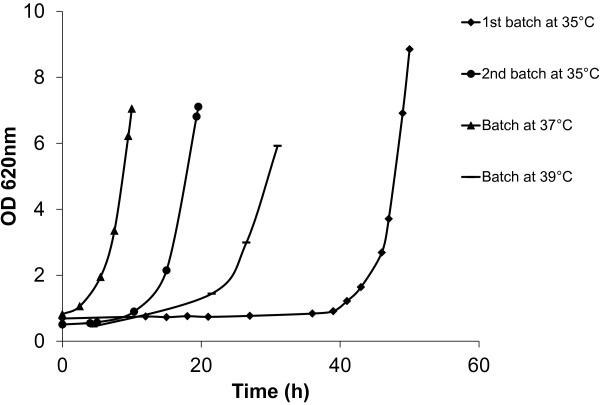
Growth of the adapted populations during serial batches with 50% (v/v) spruce hydrolysate at increasing temperatures.

Every new batch was started with an initial OD620 of 0.5 and the volume of fresh medium added was adjusted accordingly. The adaptation process was performed at different temperatures in the following order: 17 batches at 35°C, 10 batches at 37°C and 54 batches at 39°C. In the first batch cultivation at 35°C ER showed a lag phase of approximately 43 hours (Figure 1). Already in the second batch a marked reduction of the lag phase was observed, which lasted only ca. 10 hours (Figure 1). When no further improvements of the lag phase or growth rate were observed, the temperature in the bioreactor was increased to 37°C. No effects on the growth rate were observable at this temperature. Ten serial batches were performed at 37°C as a way of conditioning the culture for the next increase in temperature. When the temperature was increased to 39°C the growth rate decreased by 35% (Figure 1), indicating severe conditions of stress for the cells. As it has been previously demonstrated that there is a direct correlation between the initial biomass concentration and the rate of inhibitor conversion [11,29], some of the batch cultures at 39°C were started with lower inoculum size (OD620 ~0.2). A low initial biomass concentration was thus expected to select for cells with better detoxification capacity and/or cells with an improved tolerance to the stress caused by the inhibitors. For these particular cultivations, a longer lag phase was observed but the maximum growth rate was the same as the cultures started at a higher concentration (OD620 = 0.5). After a total of 83 sequential batch cultivations, equivalent to 280 generations approximately, the experiment was ended and the pertinence and progress of the adaptation was evaluated.

### Selection of tolerant isolates

Populations from different points in the adaptation process, including the population in the first batch, were screened on agar plates containing 50% (v/v) hydrolysate at 39°C. The cells originating from batches performed at 35°C and 37°C did not grow under these conditions. In contrast, colonies appeared after 60 to 90 hours of incubation of cells originating from batches performed at 39°C (data not shown). The fastest growing colonies were also evaluated in 50% hydrolysate at 39°C. The adapted clones were able to grow while the parental strain could not resume growth even after 55 h of incubation (data not shown). Finally, in order to ensure that the clones were genetically stable, ten sequential transfers of each clone were performed under non-selective conditions (YPD plates incubated at 30°C). After these transfers, four clones were evaluated for aerobic growth on 50% (v/v) hydrolysate medium at 39°C. Three out of the four clones tested showed a significant variability in their performance during biological replicates. Because of this, they were not considered for further characterization, and their genetic stability was not analyzed in more detailed. The last clone that showed a maintained growth phenotype and good reproducibility after the serial transfers was named ISO12 and selected for further characterization.

### Growth and fermentation performance of the adapted strain ISO12

**Figure 2:**
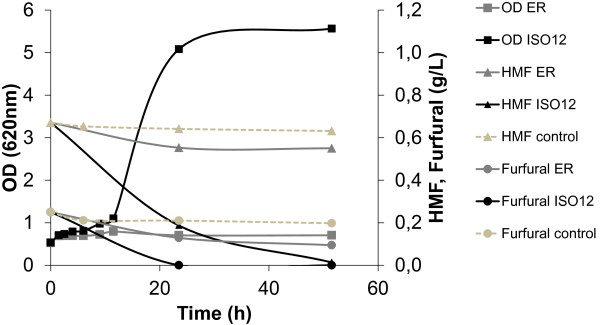
**Growth of ER and ISO12 in shake flasks with 50% (v/v) spruce hydrolysate at 39°C.** The profiles of growth and *in vivo* conversion of HMF and furfural (shown as disappearance of the compounds) are presented. Cells were pre-grown on YPD at 30°C. The experiment was performed in duplicate and the figure shows a representative profile for each strain (Dashed lines: medium alone as control).

**Figure 3:**
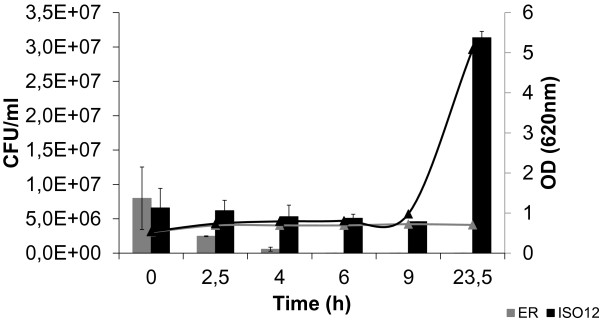
**Cell viability and OD620 values for ER and ISO12 during growth on 50% (v/v) hydrolysate at 39°C.** The experiment was performed in biological duplicates and the figure shows the data of one representative profile for each strain.

The evolved strain ISO12 and the parental strain ER were compared for aerobic growth, furaldehyde reduction and cell survival at 39°C in 50% (v/v) hydrolysate-containing medium. Without any pre-adaptation and using an initial inoculum of 0.5 (OD620), there was no increase in OD620 after 50 hours for ER, whereas ISO12 showed significant growth (Figure 2). The absence of growth by ER correlated with a complete loss of viability within the first 6 hours (Figure 3). In contrast, ISO12 maintained a high fraction of viable cells during the first 6 hours, and a 5-fold increase in cell number was observed after 23 h. In parallel, and in contrast to the parental strain, ISO12 was able to completely reduce the furaldehyde inhibitors HMF and furfural that were present in the lignocellulosic hydrolysate medium (Figure 2).

**Figure 4:**
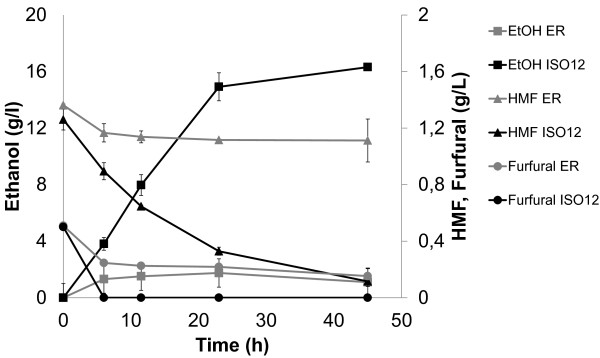
**Ethanol profile for ER and ISO12 obtained in small vials with 100% hydrolysate at 39°C.** The profiles of *in vivo* conversion of furfural and HMF (shown as disappearance of the compounds) are also included. Initial biomass was about 1 g/l cdw. Cells were pre-grown on YPD at 30°C. The values are the average of at least two independent experiments. The bars represent the deviation from the average.

Fermentation of undiluted, non-detoxified 100% spruce hydrolysate was evaluated under oxygen limited conditions using YPD-grown cells of ISO12 and the parental strain ER at 39°C. With an inoculum of 0.83 ± 0.04 g cdw/l, ISO12 was able to ferment the hydrolysate and reached a final ethanol concentration of 16 g/l (Figure 4), equivalent to a yield of 0.38 g ethanol. g hexoses^-1^. On the other hand, with 0.77 ± 0.03 g cdw/l ER barely reached a final concentration of 2 g/l ethanol, without any further increase after 24 h of cultivation. As in diluted hydrolysate, both furaldehydes were completely reduced during the fermentation by ISO12 but not by ER (Figure 4).

A possible improved tolerance towards the organic acids found in the hydrolysate was assessed by comparing growth and fermentation capacity of ER and ISO12 in mineral medium supplemented with 3.8 g/L formic acid, 5.6 g/L acetic acid and 1.4 g/L levulinic acid. Nevertheless, no significant difference was observed between the strains (data not shown).

### Evaluation of individual stress factors

**Figure 5:**
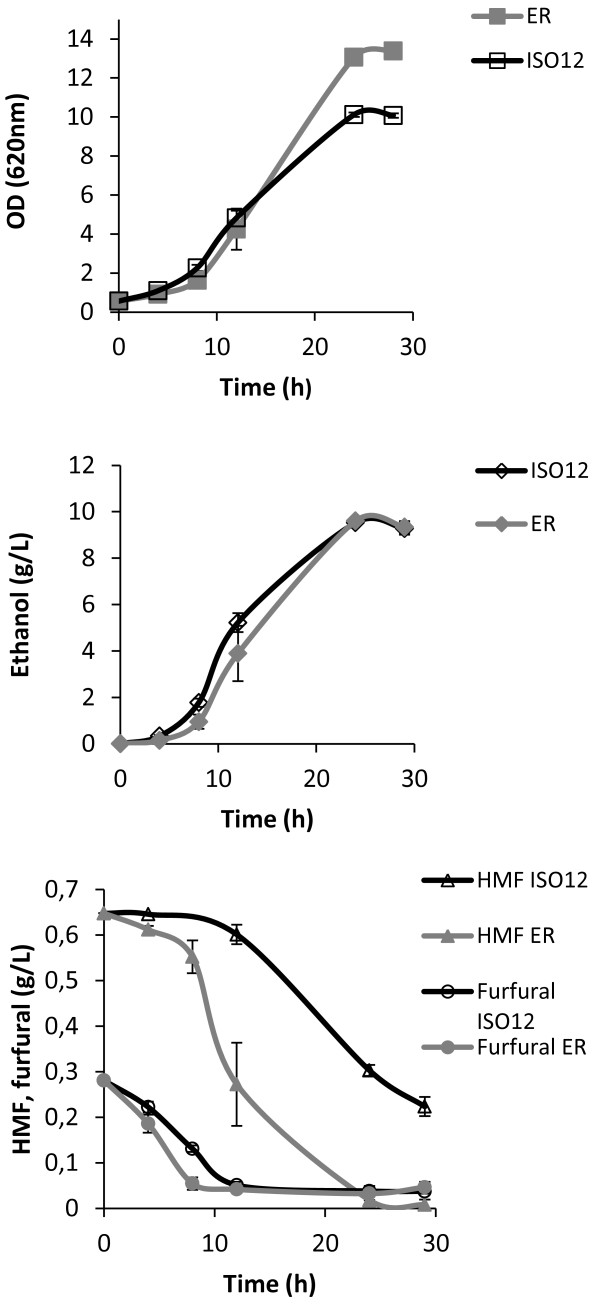
**Growth of ER and ISO12 in shake flasks with 50% (v/v) hydrolysate at 30°C.** The profiles of growth, ethanol and *in vivo* conversion of furfural and HMF (shown as disappearance of the compounds) are also presented. Measurements were obtained from two independent experiments. The bars represent the deviation from the average.

In order to elucidate whether the better performance of ISO12 was due to an increased fitness towards one or both stresses, tolerance to inhibitors and temperature was tested separately. Inhibitor tolerance was evaluated by measuring growth of the parental and adapted strains in 50% (v/v) hydrolysate at 30°C instead of 39°C (Figure 5). Similar final ethanol levels were achieved for both strains although a higher final OD620 was obtained with the parental strain ER. Since the two strains displayed similar OD620 to cell dry weight ratio (data not shown), this higher final OD of the parental strain indicated a higher biomass formation. The *in vivo* capacity to reduce furfural was similar for both strains, whereas HMF conversion was markedly and unexpectedly lower for the adapted strain ISO12. Further measurements of *in vitro* NAD(P)H-dependent HMF and furfural reductase activity indicated that the NADPH-dependent reduction activity was significantly lower for both substrates in ISO12 than in ER (Table 2). The NADH-dependent furfural activity was, however, similar in both strains at 30°C (Table 2). None of the strains showed NADH-dependent HMF reductase activity. The NADPH-dependent furfural reduction activity in ISO12 was also evaluated at 39°C, but no significant change was observed compared to the activity measured at 30°C. A NADPH-dependent HMF reduction activity, however, was not detected (Table 2). For the parental strain the specific activity for both substrates using both co-factors was negatively affected by the increase in temperature (Table 2).

**Table 2:** Furfural and HMF reduction activity in crude cell extracts of Ethanol Red and ISO12

**Temperature**	**Strain**	**Specific activity on furfural (mU/mg)**		**Specific activity on HMF (mU/mg)**	
		**NADH**	**NADPH**	**NADH**	**NADPH**
30°C	Ethanol Red	792 ± 247	479 ± 115	ND	514 ± 81
	ISO12	677 ± 263	45 ± 9	ND	26 ± 4
39°C	Ethanol Red	352 ± 202	179 ± 21	ND	200 ± 23
	ISO12	372 ± 137	31 ± 7	ND	ND

The activities were measured using NADH or NADPH as co-factor. The values are averages of at least two independent measurements and standard deviations are presented.

**Figure 6:**
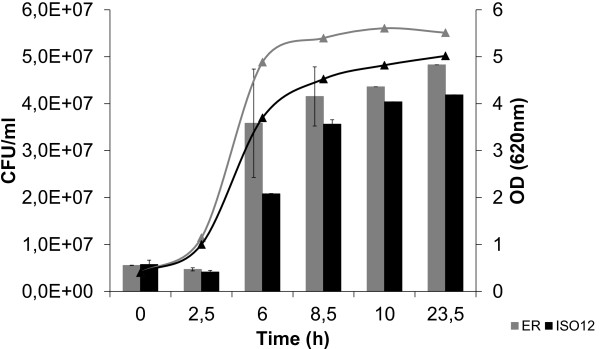
**Cell viability and OD620 values of ER and ISO12 when growing on defined mineral medium at 39°C.** The experiment was performed in biological duplicates and the figure shows the data of one representative profile for each strain.

To compare the performance of both strains in relation to heat-stress, two different experiments were carried out: i) evaluation of growth in the absence of inhibitors and ii) heat-shock experiment. The growth of both strains in the absence of hydrolysate-derived inhibitors, i.e. in defined mineral medium, was compared at 39°C. Both strains were able to grow under these conditions, but ER showed a slightly higher growth rate and final biomass concentration than the evolved strain (Figure 6). The heat-shock experiments were performed at 52°C for 20 minutes. The survival ratio was less than 10% for both strains. Still, the percentage of viable cells of ISO12 after the heat shock was around 3-fold higher (*P* = 0.02; *n* = 2) than that of the parental strain (i.e. 3.1% of viable cells for ISO12 vs. 0.95% of viable cells for ER). This result indicated that the thermotolerance of ISO12, defined as the surviving fraction of cells after incubation at a temperature that normally does not permit growth [30], was indeed improved during the evolution process.

## Discussion

The current study describes the successful use of evolutionary engineering to create a stable industrial strain that efficiently ferments non-detoxified lignocellulosic hydrolysate at higher temperature, with a resulting ethanol yield of 0.38 g ethanol/g hexoses. The evolved strain ISO12 could also grow aerobically in the presence of a combined amount of inhibitors at 39°C without requiring any pre-adaptation step.

The parental industrial strain Ethanol Red, a robust ethanol-tolerant strain, was developed to ferment sugars from grain mashes at relative high temperature, with an optimal fermentation temperature of 34°C. Still, changes in lag phase and growth rate of ER in 50% (v/v) spruce hydrolysate compared to defined mineral medium clearly indicated severe inhibitory effects of the different compounds released during biomass pre-treatment, which emphasized the importance of selecting yeast strains for combined stress tolerance. Since many of the inhibitors commonly found in spruce hydrolysate act synergistically [31], the use of the liquid fraction of the pre-treated biomass for the evolution was expected to lead to a greater resistance of the yeast towards this cocktail of inhibitors. Previous adaptation experiments using either single inhibitors or a mix of them at optimal yeast temperature have indeed led to the development of strains with higher capacities for conversion of furfural and HMF [11,12]. In parallel, identification and further overexpression of various furaldehyde reductases, mostly NADPH-dependent, in *S. cerevisiae* strains also resulted in faster degree of conversion of furaldehydes in both defined mineral medium and hydrolysate [32,33]. In our case, the disappearance of furfural and HMF from the medium indicated that furaldehyde detoxification at 39°C was indeed actively carried out by ISO12 but not by the parental strain ER. However ISO12 did not show enhanced furaldehyde reductase activities, but rather lower *in vitro* NADPH-specific HMF and furfural reductase activities (and unchanged NADH-dependent furfural activities) at both 30°C and 39°C. However, since ISO12 had the capacity of remaining viable under the stress conditions, it could perform the detoxification of the medium although with lower reductase activities. For ER, on the other hand, it is highly probable that its limited *in vivo* reduction capacity was rather due to a rapid loss of viability and overall reduced metabolic activity under the conditions of combined stresses. Alternatively, ISO12 may have acquired robustness towards other types of inhibitors, particularly phenolics, during the evolution process. Unfortunately, the diversity of this class of inhibitors, together with the limited methods for their analysis and quantification, did not enable us to assess their inhibitory effect [34].

The decrease in NADPH-dependent reductase activities displayed by ISO12 may have occurred as a trade-off situation during the evolution process. Trade-off events are very common during evolutionary experiments, and are seen as the “pay-off” for the improved traits [16,35]. For this particular case, we hypothesized that the evolution experiment may have triggered the selection of strains with reduced NADPH usage for non-essential reactions because such trait may become beneficial during heat stress as the dinucleotide NADPH is required as reductive power during the synthesis of amino acids and fatty acids [36]. Under the adaptation conditions, the thermal stress is expected to require additional readjustments of metabolic pathway fluxes, including a higher turnover of proteins, and therefore a higher demand of available NADPH for biosynthetic purposes. Furthermore, a down-regulation of non-crucial enzymes that use NADPH as cofactor would result in higher availability of the cofactor for other more critical reactions, such as the use of NADPH by the thioredoxin and glutathion/glutaredoxin systems to defend the cells against stress caused by reactive oxygen species (ROS) [37]. Under the conditions of aerobic adaptation, the cells would be under high ROS stress since heat, oxygen and at least furfural (among the inhibitors present in the hydrolysate) have been associated with increased amounts of ROS in the cell [38]. Another less obvious, but plausible, explanation of a higher requirement of NADPH would be an increased demand for NADPH-demanding sphingolipid synthesis [39] since it has been demonstrated that heat stress can induce *de novo* synthesis of sphingolipids, which contributes to an increase in thermotolerance [40]. Altogether, this would lead to a readjustment in the levels of NADPH-dependent reactions, and such readjustment would remain even when the heat stress was no longer present.

The lower growth rate and final biomass concentration showed by ISO12 when growing in mineral medium at 39°C was also observed during growth on 50% (v/v) spruce hydrolysate at 30°C (Figure 5), and even during growth on YPD medium at 30°C (data not shown). Although such slower growth could be interpreted as a trade-off situation during the adaptation process, previous studies suggest that the reduction in maximum specific growth rate could be one of the mechanisms behind the better ability of ISO12 to survive a thermal challenge; since it has been observed that slower growth predicts a higher resistance to heat stress [41,42].

A significantly increased thermotolerance was demonstrated for ISO12 as compared to ER using heat-shock experiments. However, this feature was not noticeable when growing strains at 39°C in the absence of lignocellulosic inhibitors. It is well known that the composition of the growth medium (among other factors) affects the temperature response profiles of yeast growth [43]. We therefore suggest that the hydrolysate-derived inhibitors, such as the highly reactive phenolic compounds, reduce the maximum permissible temperature for growth to a value below 39°C, and that the acquired changes in ISO12 allowed the strain to overcome the synergistic effects of inhibitor and thermal stresses. The higher robustness of ISO12 towards high temperatures would notably permit all the reactions necessary for growth, including inhibitor detoxification, to be functional.

## Conclusions

Through an evolutionary engineering approach for improvement of combined thermotolerance and tolerance towards hydrolysate-derived inhibitors the stable industrial strain ISO12 was obtained. ISO12, contrary to the parental strain, is able to grow and ferment non-detoxified lignocellulosic hydrolysate at higher temperature. Development of more robust strains such as ISO12 may contribute to improve ethanol production in tropical countries or when using lignocellulosic raw materials at higher process temperature (e.g., in SSF). It also serves as a potential platform strain for further metabolic engineering, such as the introduction of the pentose fermentation capacity. Finally, the ISO12 strain represents an interesting tool to analyze possible regulatory mechanisms that have emerged as a result of combined stresses for relatively long periods.

## Methods

### Strain

The industrial *Saccharomyces cerevisiae* strain Ethanol Red [Fermentis, S.I. Lesaffre] (ER) was used as a starting point for the evolution. The strain was maintained on YPD agar plates (10 g/l yeast extract, 20 g/l peptone, 20 g/l glucose and 15 g/l agar).

### Hydrolysate

The liquid fraction of spruce hydrolysate was obtained from SEKAB, Sweden. The hydrolysate was diluted to different concentrations (% v/v) with defined mineral medium [44] according to the experimental requirements. The major components of the hydrolysate before dilution were (g/l): 15.8 glucose, 22.3 mannose, 4.5 galactose, 3.8 arabinose, 10.7 xylose, 3.8 formic acid, 5.6 acetic acid, 1.4 levulinic acid, 1.5 HMF and 0.5 furfural. The pH of the hydrolysate was adjusted to 5.2 with 8 M KOH prior to use.

The evolution experiment and the strain characterization experiments were performed using 50% hydrolysate. This medium was buffered with 50 mM potassium hydrogen phthalate buffer (pH 5.2) and supplemented with hexose sugars to mimic the sugar composition in 100% hydrolysate.

### Evolution experiment

The initial condition for the evolution experiment was determined by evaluating the growth of ER in different concentrations of hydrolysate at 35°C. Cell growth was followed by measuring the OD of the culture broth at 620 nm using a U-1800 spectrophotometer (Hitachi, Tokyo, Japan). For this, overnight cultures in YPD were used to inoculate 250 ml flasks containing 25 ml of the respective medium (0, 25, 50 and 80% hydrolysate (v/v)), at an initial OD620 of 0.5. The flasks were incubated at 200 rpm and OD620 measurements were used to determine the duration of the lag phase and the specific growth rate for each condition.

The evolution of ER was performed as aerobic serial batches in a 1.4 L Multifors bioreactor (Infors, Switzerland) with a working volume of 700 ml. The agitation and aeration were set at 300 rpm and 1 vvm respectively. The pH was kept constant at 5.2 by addition of 1 M NaOH. For inoculum preparation ER was cultivated at 150 rpm and 30°C in 500 ml shake flask containing 50 ml YPD medium until it reached late exponential phase. An appropriate volume of this culture was used to inoculate the bioreactor to an initial OD620 of 0.5. The supernatant of the broth was used as a reference in each OD620 measurement. When the culture reached mid-log phase, approximately 90% of the broth was pumped out of the bioreactor and replaced with new medium. Every three batches, samples of the population were collected and stored at -80°C in 20% (v/v) glycerol solution. The initial temperature of the serial batches was set to 35°C which was subsequently increased to 37°C and finally to 39°C.

### Selection and characterization of isolates

Glycerol stocks of populations from selected points in the adaptation were streaked on solid 50% hydrolysate medium and incubated at 39°C. The first colonies that appeared were selected for further analysis.

a) *Aerobic growth*

The experiments were carried out as described above except that the growth was evaluated using 50% hydrolysate in 500 ml shake flasks containing 50 ml medium. Growth was evaluated at 30°C and 39°C for each isolate and samples were also collected for analysis of product formation by HPLC.

b) *Cell viability*

Cell viability was determined during cultivation at 39°C in both defined mineral medium and 50% hydrolysate medium using the plate count method [45]. Briefly, serial dilutions of the cultures at different time points were plated on solid YPD and incubated at 30°C. After 48h, colonies were counted and viability is reported as CFU/ml.

c) *Anaerobic fermentation*

The anaerobic fermentation performance was evaluated in sealed serum flasks containing 100% hydrolysate supplemented with 1 g/l yeast extract, 0.5 g/l (NH_4_)_2_HPO_4_ and 0.025 g/l MgSO_4_ . 7H_2_O. Cells pre-grown in YPD over-night were harvested by centrifugation, washed with deionized water and resuspended in the hydrolysate medium to a concentration of ca. 1 g/l cell dry weight (cdw). Oxygen-limited conditions were obtained by adding a top-layer of mineral oil and having a small head space in the flasks. A cotton-filled syringe was inserted through the rubber stopper using a needle to avoid accumulation of gas inside the flasks. The flasks were incubated in a water bath at 39°C and the agitation was maintained at ca. 150 rpm using a magnetic stirrer.

d) *Analysis of fermentation products*

Ethanol, HMF and furfural concentrations were determined by High Performance Liquid Chromatography (HPLC), using Waters HPLC system (Milford, MA, USA). An Aminex HPX-87H ion exchange column (Bio-Rad, Hercules, CA, USA) was used for separation and a refractive index detector (RID-6a, Shimadzu, Kyoto, Japan) was used for detection. The mobile phase was 5 mM H_2_SO_4_ at a flow rate of 0.6 ml/min and the column temperature was 45°C. Cell dry weight of the inoculum was determined by filtering the sample through a dried and pre-weighed membrane with 0.45 μm pore-size (Pall Corporation, NY, USA), washing with distilled water, and drying for 8 minutes in a microwave (350W).

### Heat shock experiments

Overnight YPD-grown cultures were centrifuged (3,220 rcf; 5 min; 4°C) and washed with sterile water. Cells were resuspended in cold YP and incubated for 30 minutes at 30°C under agitation (180 rpm). After incubation, cells were centrifuged as above and resuspended in cold YP medium. For each strain, 0.5 ml aliquots of resuspended cells were transferred to two microfuge tubes: one tube was maintained on ice (control) and the other was incubated in a water bath at 52°C for 20 min followed by incubation on ice. Finally, serial dilutions of the cells were plated on solid YPD and incubated at 30°C. After 48 h the colonies were counted and the percentage of survival was obtained by comparing the CFU:s obtained after the heat-shock with the control.

### Enzymatic assays

Cells were cultivated in 250 ml shake flasks containing YPD or 50% hydrolysate. Cell-free protein extracts were prepared using Y-PER according to the manufacturer’s instructions (Pierce, Rockford, IL, USA). Total protein concentration was measured using Coomassie dye reagent and bovine serum albumin as standard (Thermo Scientific, Rockford, IL, USA). Reductase activity was determined as previously described [46], using either furfural or 5-(hydroxymethyl)furfural as the substrate; NADH or NADPH as cofactor, and potassium phosphate (100 mM, pH 7.0) as buffering agent. Enzyme activities were recorded at 340 nm using Ultrospec 2100 pro spectrophotometer (Amersham Biosciences, Uppsala, Sweden) at 30°C and Hitachi U-2000 spectrophotometer (Hitachi, Tokyo, Japan) at 39 ±0.3°C.

## Abbreviations

SSF: Simultaneous saccharification and fermentation; ER: Ethanol Red; HMF: 5-(hydroxymethyl) furfural; OD: Optical density; YPD: Yeast extract peptone dextrose; Cdw: Cell dry weight; ROS: Reactive oxygen species; HPLC: High performance liquid chromatography; CFU: Colony-forming unit.

## Competing interests

The author(s) declare that they have no competing interests.

## Authors’ contributions

VWS participated in the design of the study, performed the experiments and drafted the manuscript. MFG conceived the study and revised the manuscript. All authors read and approved the final manuscript.
